# The Banquet A. D. M. S. and Election of Officers

**Published:** 1891-12

**Authors:** 


THE BANQUET.

The session held till 9 p. m., when the members were invited by the Austin District Medical Society to a splendid banquet, where the popping of bottles punctuated and emphasized the many good things that were said when the toasts came on.

The first toast was proposed by Dr. Swearingen—"To the Medical Department of our State University." Professor West responded. The medical school is fairly launched, he said, and is under full headway. Though the class is comparatively small—as was to be expected from the length of the courses required, three graded courses of seven months each,—the examination for matriculation—the full fees for the courses—the high standard fixed for graduation—all felt encouraged; and the faculty, chosen with much circumspection, were all earnest and enthusiastic in their work. Students entered there, could have gone to quite reputable colleges, where, for one-third the expense and one-half the time, perhaps, they could have gotten through, and sailed out on the sea of practice with a diploma. But the diploma from the Texas Medical College could not be so easily obtained. He who wins it, must earn it. The standard has been fixed high, and it is the duty of every physician in Texas who feels a pride in his profession, and believes in elevating medical education, to lend his hearty coöperation; and, on behalf of the faculty, he now asks the physicians of Texas to help the cause by their unqualified support and coöperation. It is the determination to make the Texas Medical College a grand success, and worthy of the great State of Texas.

Dr. Wooten, President of the Board of Regents of the Texas

University, responded to a toast "to the University of Texas." He showed, in forcible language, the great work for education Texas has performed, and dwelt especially upon the fact that the Medical College is supported by the State, and is in no way dependent upon patronage for its success.

Dr. Blailock, at request of Dr. Daniel, responded to the toast (by Dr. Daniel) to "Our visiting friends." Drs. B. and D. were formerly colleagues in the Mississippi Medical Association. Dr. Blailock acquitted himself with his usual grace and eloquence.

Dr. Daniel responded to the usual toast, to the "Medical Press."

The occasion was one long to be remembered by all who had the good fortune to be present. We bade our guests "good bye," and promised to return their visit in the sweet bye and bye.

